# Death, organ transplantation and medical practice

**DOI:** 10.1186/1747-5341-3-5

**Published:** 2008-02-04

**Authors:** Thomas S Huddle, Michael A Schwartz, F Amos Bailey, Michael A Bos

**Affiliations:** 1 Division of General Internal Medicine, University of Alabama School of Medicine and the Birmingham VA Medical Center, FOT 720, 1530 3rd Avenue South, Birmingham, AL 35294, USA; 2 Department of Psychiatry, University of Hawaii, Department of Philosophy and Psychiatry, University of Louisville, 1106 Blackacre Trail, Austin, TX 78746, USA; 3 Director, Palliative Care, Birmingham VA Medical Center, 700 19th Street South, Birmingham AL 35233, USA; 4 Senior Scientific Advisor, Health Council of the Netherlands, PO Box 16052, 2500 BB The Hague, The Netherlands

## Abstract

A series of papers in *Philosophy, Ethics and Humanities in Medicine (PEHM) *have recently disputed whether non-heart beating organ donors are alive and whether non-heart beating organ donation (NHBD) contravenes the dead donor rule. Several authors who argue that NHBD involves harvesting organs from live patients appeal to "strong irreversibility" (death beyond the reach of resuscitative efforts to restore life) as a necessary criterion that patients must meet before physicians can declare them to be dead. Sam Shemie, who defends our current practice of NHBD, holds that in fact physicians consider patients to be dead or not according to physician intention to resuscitate or not.

We suggest that criteria for a concept are not necessarily truth conditions for assertions involving the concept. Hence, non-heart beating donors may be declared dead without meeting the criterion of strong irreversibility even though strong irreversibility is implied by the concept of death. Our perception that a concept applies in a given case is determined not by the concept itself but by our necessary skill and judgment when using it. In the case of deciding that a patient is dead, such judgment is learned by physicians as they learn the practice of medicine and may vary according to circumstances. Current practice of NHBD can therefore be defended without abandoning death as an empirical concept, as Shemie appears to do. We conclude that the dead donor rule continues to be viable and ought to be retained so as to guarantee what the public most cares about as regards organ donation: that physicians can be trusted to make determinations of eligibility for organ donation in the interests of patients and not for other purposes such as increasing the availability of organs.

## Editorial

Physicians and ethicists are once again debating definitions of death. The practice of non-heart beating organ donation (NHBD) appears to many to violate the dead donor rule, which requires that death precede organ donation and that living patients not be killed for organ procurement [1]. Controlled NHBD involves withdrawing support for brain-injured patients in the operating room and harvesting their organs shortly after cardiopulmonary arrest. The interval between arrest and a declaration of death is as short as two minutes in Pittsburgh, where NHBD was developed in 1992 [2], or as long as five to ten minutes in most of Europe. It has been argued that such patients do not meet valid criteria for death shortly after cardiopulmonary arrest and that controlled NHBD therefore amounts to harvesting organs from live patients.

Complicating the discussion are increasing misgivings about the concept of brain death. While that concept has been generally accepted as a clinically useful tool since its introduction by the ad hoc Harvard Committee to examine the definition of brain death [3], a growing body of knowledge about severely-brain injured patients has challenged its coherence. Philosophical justifications of a brain death criterion for death have relied upon a notion of life involving an essential integration of bodily functions provided by the brain, the absence of which is death. Studies of brain dead patients, some of whom have been kept "alive" for long periods, suggest that in fact the brain is not essential to many aspects of organismal integration and that bodily integration may not be best thought of as localized to a particular organ [4]. If this is so, the notion of brain-mediated bodily integration does not offer a reason to suppose that brain dead patients are really dead. Another layer of complexity has been added by a growing awareness of multiple points at which a patient might plausibly be declared dead. Shewmon explicates such points relevant to both heart and brain definitions of death (table 1).

**Table 1:** 

E1	Final apnea
E2	Final asystole
E3	Loss of potential for cardiac autoresuscitation
E4	Loss of potential for interventional resuscitation
E5	Onset of permanent loss of consciousness
E6	Loss of potential for recovery of consciousness
E7	Irreversible loss of all brain function

Adapted from DA Shewmon, The dead donor rule: lessons from linguistics. Kenn Inst Ethics J 14(2004): 277–300.

In recent months *Philosophy, Ethics, and Humanities in Medicine (PEHM) *has hosted a lively discussion of whether NHBD patients are really dead before organ donation and of the implications of NHBD for the dead donor rule and definitions of death. Joseph Verheijde and colleagues contend that patients subjected to NHBD are dying rather than dead at the time of donation. Calling such patients "dead" amounts to a manipulation of the definition of death for purposes of furthering organ donation. Facing burgeoning technology, pluralistic societies must respect patient autonomy as well as religious, cultural and ethnic diversity and encourage public debate about organ donation and about when death should be declared. All adults would then be able to explicitly give or withhold consent to donation [5,6]. This position is seconded by David Wainwright Evans, who questions the validity of "brain death" and agrees that NHBD patients are dying rather than dead [7]. Michael Potts agrees but draws the conclusion that NHBD should be forbidden as it involves physicians in killing patients [8]. Sam Shemie defends current practice, arguing that there has never been an isolable line between life and death and that in current medical practice death is determined not only by the state of the organism but by physician intentions to resuscitate or not. That being the case, two minutes of cardiac arrest in the absence of resuscitative efforts is sufficient to declare a patient dead before organ donation [9]. Ari R. Joffe notes difficulties with the concept of brain death and contends that we must consider patients dead only after irreversible cardiopulmonary arrest. He faults Shemie for favoring "weak" irreversibility (irreversibility because of physician intention not to resuscitate) as the relevant criterion for death because that standard would have us labeling patients in identical physiological states as dead or not dead yet according to context. Only "strong" irreversibility–cardiopulmonary arrest or brain death beyond the reach of resuscitative efforts to restore circulation, breathing, or brain function (E4 or E7 in table 1)–can be the proper criterion for death. NHBD protocols are thus harvesting organs from patients who are not dead–this must be acknowledged and publicly debated [10]. Finally Bellomo and Zemperetti regard a continued dependence upon a cardiopulmonary criterion for death as essential for societal acceptance of organ donation, although they regard any such criterion as an arbitrarily drawn line across the death process–since "hypertechnological medicine" has left us without an objectively identifiable border between life and death [11].

For many, non-heart-beating organ donation has sharpened a dilemma that we must face in using any concept. Our concepts must be either descriptive or evaluative. Either we observe death and only then declare its presence; or we construct death, as it were, by declaring it according to our purposes in doing so. In the eyes of many the practice of NHBD has moved us into the latter posture, as we appear to be harvesting organs from patients who are dead more by stipulation than by empirical confirmation. The concept of death must either remain descriptive or be recognized as unequivocally evaluative. We must acknowledge that NHBD involves taking organs from the living if our concept of death is to remain descriptive. If, on the other hand, we are willing to give up death as an empirical concept we can retain NHBD and the dead donor rule by changing the definition of death so that NHBD falls on the far side of the line between life and death, thus altering our concepts to fit our practice [12].

The former option is probably the more attractive to most physicians, trained as they are in a scientific tradition according to which empirical concepts must be rigidly applied. As Bernat states, life and death are non-overlapping, dichotomous, and jointly exhaustive states [13]; death is therefore an event, not a process; and if medical progress successfully teases out previously invisible points at the boundary between life and death then one such point will best fit the conceptual line that must continue to divide the two states. If non-heart-beating donors fall on the near side of that line, then we must accept that they are not yet dead at the time of organ donation. Hence Truog's proposal that we permit organ donation before death given informed consent and adequate safeguards against abuse [14]. The alternative, changing our concept of death to reconcile the practice of NHBD with our conviction that we must not take organs from patients before they are dead, seems an arbitrary gerrymandering of what ought to remain an empirical concept.

We would contend, however, that our concepts do not divide neatly between the fixed empirical and the consensual evaluative; that the dilemma, in fact, is illusory. Any intelligible concept must be governed by impersonal public standards. But empirical concepts can evolve or change without necessarily succumbing to unacceptable subjectivity. Indeed, they must do so if they are to keep up with changes in our practices or changes in our knowledge of the world. This is to say, contra Bernat, that our empirical concepts are necessarily "open" rather than fixed and immutable [15]. New discoveries or practices can alter them–a common enough phenomenon in the history of science. A better view of points near the transition from life to death afforded by contemporary medical practice does not necessarily mandate choice of one such point as the "real" boundary between the two states. If we have previously regarded points ranging from E1 to E7 as the relevant boundary, our concepts are potentially compatible with any of them, or, perhaps, with all of them depending upon context [16].

While our empirical concepts may be "open," our concept of death certainly retains the notion of "strong" irreversibility. Nothing in recent medical practice has altered our continued conviction that death is not reversible by medical means. But we still may not have to choose between declaring non-heart-beating donors alive and gerrymandering the concept of death. Those who suppose otherwise cite the hitherto unobserved points along the trajectory from life to death brought within our view by contemporary medical practice. Bringing our concept of death to bear upon this newly visible part of that trajectory, they conclude that the boundary between life and death must fall at a point beyond which our most potent technology can no longer achieve continued life–so that "strong" irreversibility is required before a patient may be declared dead. This is to confuse aspects of the concept of death, which certainly include "strong" irreversibility, with the kind of evidence we require in given situations to conclude that a patient is dead, which may vary; to confuse aspects of a concept with fixing its extension.

Wittgenstein and his interpreters argue persuasively that criteria for a concept do not always determine our view of when a given possible instance falls under it. Criteria are sometimes truth conditions for assertions and sometimes not [17,18]. In the case of the concepts of life and death, this is confirmed when we consider the death of patients in the hospital with and without do-not-resuscitate orders on the chart. Patients in the hospital for whom do-not-resuscitate (DNR) orders are written, should they die as inpatients, do so in the traditional way–their death is marked by the visible cessation of respiration, more or less coincident with cardiac arrest. They are pronounced dead by physicians shortly thereafter, often surrounded by family and friends as might happen at home. Patients without DNR orders who stop breathing in the hospital are regarded differently; they are not dead, or, at least, not dead yet. After twenty minutes or more of vigorous resuscitative efforts they will be pronounced dead if they do not respond; or, perhaps, their circulation and ventilation restored with more or less aid from pressors and mechanical ventilation, they will still be alive. Is it the case then that patients with and without DNR orders, who may be in physiologically similar states shortly after respiratory or cardiac arrest, are arbitrarily regarded as dead or not according to physician intention to resuscitate or not? No; a physician declares the patient with DNR orders to be dead shortly after cardiopulmonary arrest because she most likely is, in fact, dead. Most resuscitative efforts are unsuccessful, suggesting that most patients in fact meet the criterion of strong irreversibility very soon after cardiorespiratory arrest. As to the patient without DNR orders who arrests, physicians suspend judgment as to life or death until resuscitative efforts have either succeeded or failed–if they succeed, the patient has, of course, not died. If they fail, we say that the patient died at some indeterminate point between cardiac arrest and the cessation of resuscitation.

We make a diagnosis of death shortly after cardiorespiratory arrest in the DNR patient not because we can make the diagnosis with absolute certainty within five minutes thereafter, but because we are willing to tolerate some diagnostic imprecision in the context of a passage from life to death that we have determined not to interrupt. In the case of the patient without DNR orders who dies unexpectedly, we apply a more rigorous test by withholding a declaration of death until resuscitative efforts have failed. We do so having judged that in such cases, a relatively favorable prognosis warrants the attempt at reversing the arrest that we then make with our resuscitative efforts. This variable readiness to make use of a clinical category is of a piece with our usual practice in medicine. We make diagnoses and label patients more or less readily depending upon the implications of diagnostic error and upon the consequences for treatment and prognosis conferred by the diagnosis. The degree of certainty we may require for a given classificatory act will vary according to what we will do when a diagnosis is made.

How much certainty ought we to require before we consider the non-heart-beating organ donor to be dead? We would contend that requiring absolute certainty that a donor cannot be resuscitated before declaring death is not warranted by the clinical context of NHBD. We confirm the death of organ donors prior to donation to protect their interests, and, secondarily, to maintain public trust in organ donation. Just as in the case of the DNR patient, we have determined not to interrupt the organ donor's passage from life to death when we withdraw ventilatory support. Within a few minutes after cardiorespiratory arrest the donor is almost certainly dead; there is no practical reason for us to delay a determination of death as we might for a patient whose more favorable prognosis warranted vigorous resuscitation.

We would thus conclude, with Shemie, that NHBD does not violate the dead donor rule. Contra Shemie, however, death remains an empirical concept and is independent of physician intention. Life and death are dichotomous states, although the boundary between them may be hazy given current medical practices. In spite of that haziness, determinations of death are not arbitrary; they are more or less precise determinations of an organism's state based upon valid concepts skillfully deployed by physicians in the interests of patients. Physicians necessarily exercise judgment in diagnosing death and such judgment may vary systematically in differing clinical contexts. If we presume that the ability to recognize instances of a concept is tightly bound to the aspects of that concept–so that we become a vehicle, as it were, of the concept of death as we categorize patients as dead or not–such variance may make us acutely uncomfortable. This presumption, we suggest, is why an intensivist unprepared to recognize death in salvageable patients in the intensive care unit after two minutes of cardiorespiratory arrest might believe herself constrained not to recognize it after two minutes of arrest in any patient.

But the ability to recognize death is not a simple function of our linguistic capacity in the form of our possession of the concept of death. Our practice confirms that practical "know how" or judgment must supplement our concepts as we connect these to the world without. While two minutes of arrest is often too short a time for the intensivist to conclude herself to be in death's presence, it is a long time indeed to the internist or palliative care physician standing by the bedside after a patient's final agonal breath; quite long enough to conclude that the patient is dead, even if the formal declaration of death is delayed for a few minutes more. Acknowledging such variance in our classifying practices is to recognize the way in which we actually use empirical concepts such as death; and it allows us to reconcile NHBD with the dead donor rule.

That rule has played an important role in assuring the public that physicians will protect the interests of patients while practicing organ transplantation. Its opponents suggest that the public might be educated to do without a rule which relies on an equivocal concept–as our contextually variable declarations of death imply that in fact multiple concepts of death are in use, no one of which can claim priority. Such a malleable notion of death cannot then do the work required of it in regulating practices such as when to bury, transfer an estate, commence an autopsy, or harvest organs. Justifications other than death must be found for our various end-of-life practices [19]. In the United States this position has been bolstered by surveys indicating that the American public has a hazy understanding of when severely brain-injured patients are legally dead [20]. The same surveys show that under some circumstances, a proportion of the public would support pre-mortem organ donation.

If we are correct, this argument against the dead donor rule is mistaken at its core, confusing concepts and their aspects with the recognition of what falls under them. A univocal and empirical concept of death is perfectly compatible with contextual variability in its use; death may thus continue to serve as the conceptual regulator of our end-of-life practices. That the public is confused is indeed a reason to undertake public education about death and organ donation. But if the public is to continue to trust physicians, we must not extend the practice of organ donation from the dead to the living. Thus we question certain refinements of NHBD currently being introduced, such as the use of extracorporeal membrane oxygenation (ECMO) for organ donors before support is withdrawn. When ECMO is used in NHBD, organ donors are never without circulation and oxygenation before organ harvesting; thus organ procurement takes place without death having occurred on any plausible understanding of the concept. The gain of ECMO is better preservation of organs harvested with its use–at the price of placing physicians in the position of killing patients while procuring their organs. While such killing may not alter the terminus of the organ donor's trajectory, which is death in any case, the agency of physicians in the donor's death is morally problematic; it is also an important step down what might prove to be a very slippery slope. We suggest that the use of ECMO transforms NHBD into "an ignoble form of cannibalism," as Renee Fox wrote of NHBD in its early days [21]. Because we physicians must continue to do no harm, we must forego the advantages of ECMO in NHBD and continue to harvest organs only from patients who have died. But we can continue to make that determination according to clinical context.

## Competing interests

The author(s) declare that they have no competing interests.

## Authors' contributions

TSH wrote the first draft. The final manuscript was the result of multiple mutually agreed modifications by TSH, MAS, FAB and MB.
